# Supplementary material for: Analysis of Genetic Diversity and Resistance to Foliar Pathogens Based on Genotyping-by-Sequencing of a Para Rubber Diversity Panel and Progeny of an Interspecific Cross
Source: Plants (Basel). 2022 Dec 7;11(24):3418. doi: 10.3390/plants11243418 (PMC9781018; doi:10.3390/plants11243418)
Supplement: Supplementary file 1 [file plants-11-03418-s001.zip › New folder/plants-1998018 Supplementary -1-1.pdf]

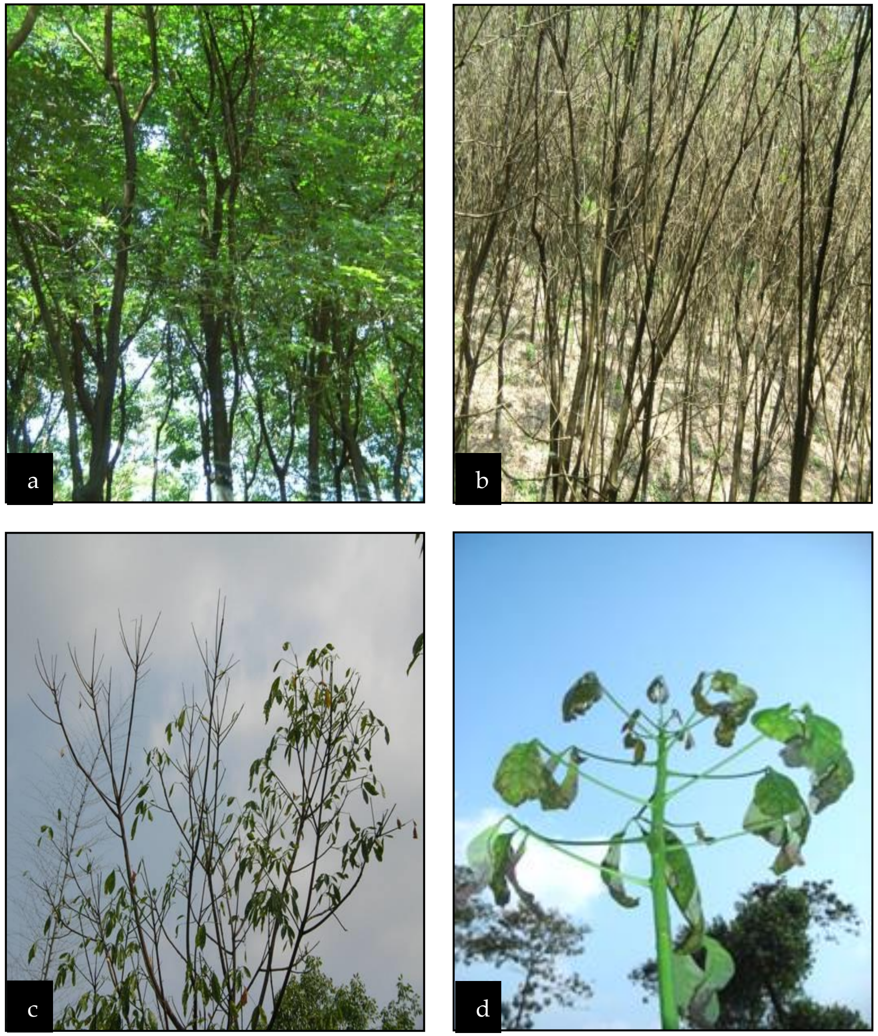

Figure S1. A healthy rubber plantation with dense canopy (a); mature trees infected with *Phytophthora* spp. causing abnormal leaf fall disease (b); young refoliating leaves showing typical symptoms of infection with *Corynespora cassiicola* causing Corynespora leaf fall disease (c) and shriveling of tender leaves infected with *Colletotrichum* spp. causing Colletotrichum leaf disease (d).

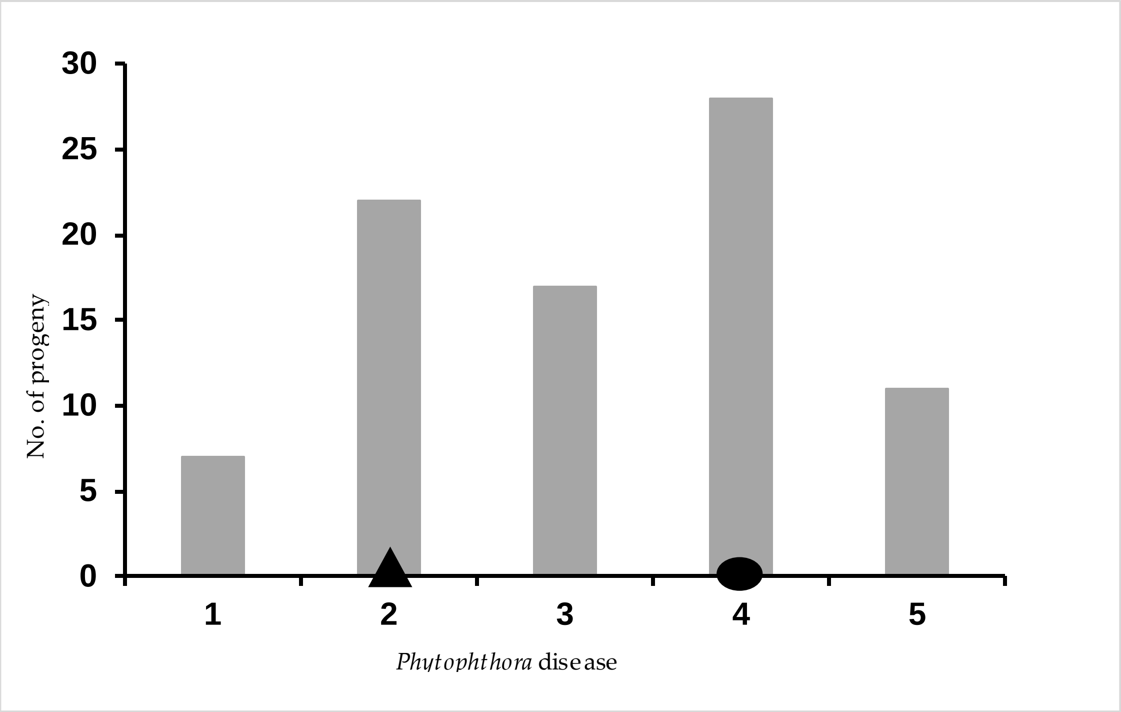

Figure S2. *Phytophthora* disease severity measured by the size of lesion. Lesions caused by *Phytophthora meadii* were assessed with 85 progeny from a cross between *H. brasiliensis* and *H. benthamiana*. Five resistance categories are indicated as highly resistant (1), resistant (2), moderately resistant (3), susceptible (4), and highly susceptible (5). Disease severities of the resistant (*H. benthamiana*) and susceptible (*H. brasiliensis*) parents are indicated by triangle and circle, respectively.

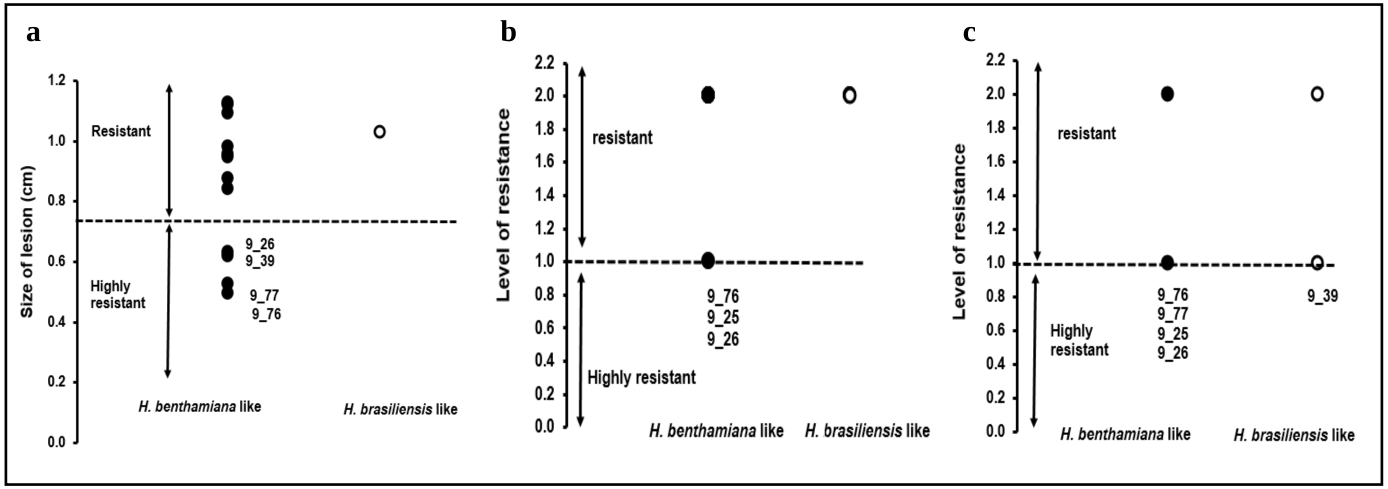

Figure S3. *Phytophthora* (a), *Corynespora* (b) and *Colletotrichum* (c) disease resistance of progeny based on KASP marker results. Level of resistance measured as size of lesion (a) rate of wilting in the leaf (b and c) caused by the resistant parent (*H. benthamiana*) is marked with a spotted line in black. The progeny (9_39) that had *H. brasiliensis* like genotype seems to carry resistance trait.

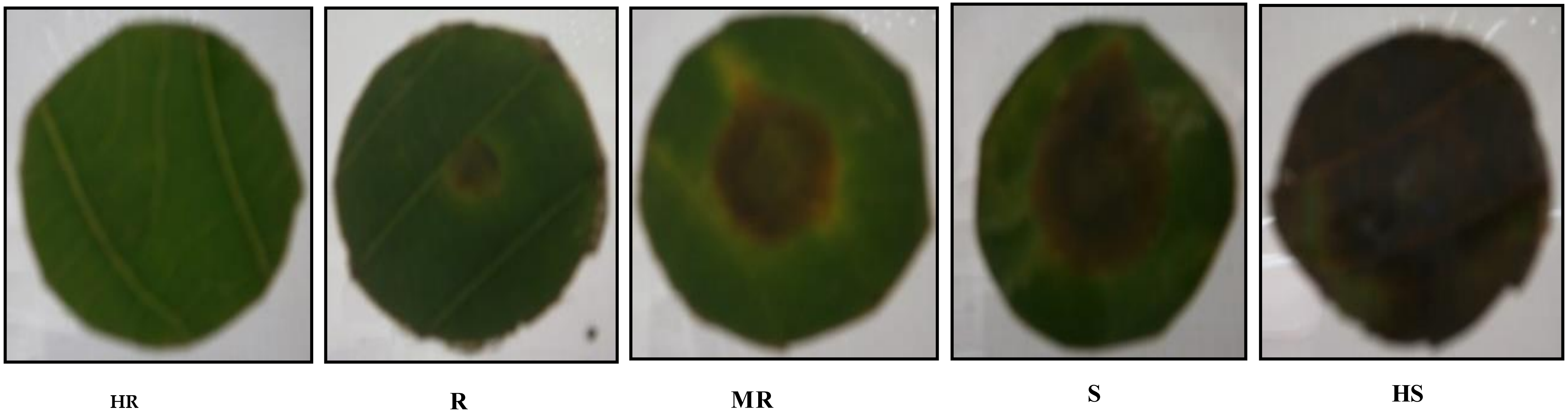

Figure S4. Assessment for *Phytophthora* disease responsein progeny population derived from a cross between *H. brasiliensis* and *H. benthamiana*. The degree of resistance is categorized as highly resistant (HR), resistant (R), moderately resistant (MR), susceptible (S) and highly susceptible (HS)based on size of lesion produced on leaf after 96 h of infection with zoospore suspension

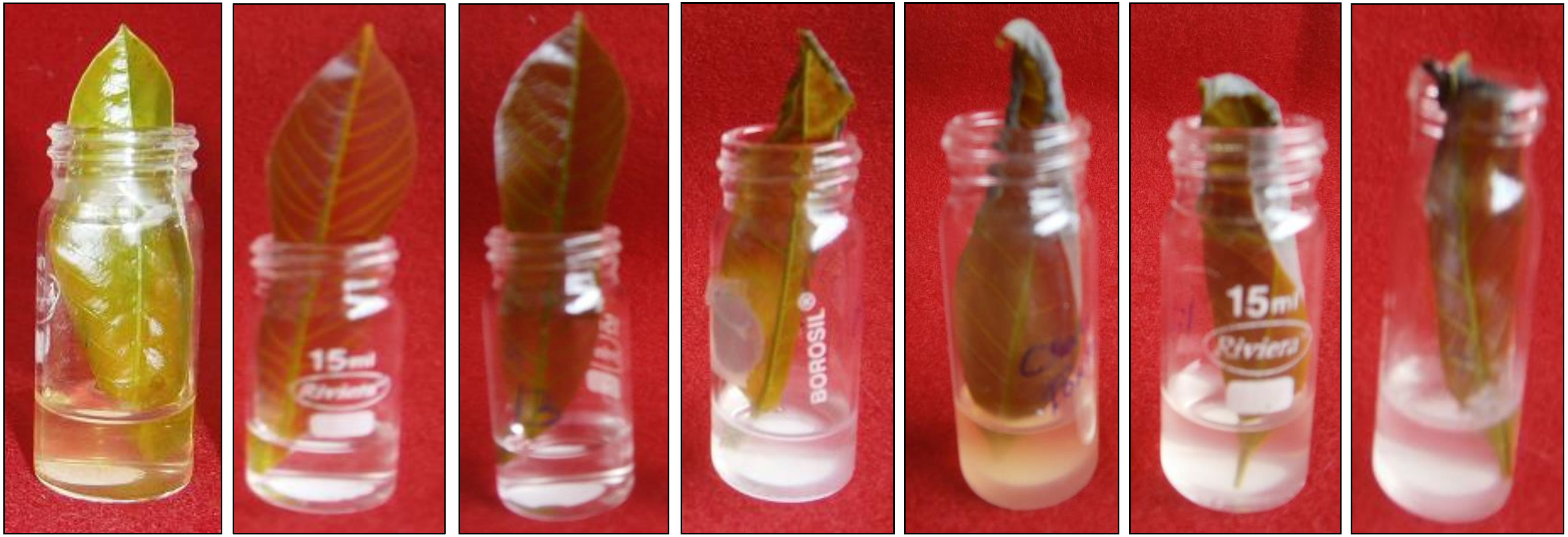

Control 1 | **Control 2** | **1** | **2** | **3** | **4** | **5**

Figure S5. Toxin-based screening for disease resistance to *Corynespora cassiicola*. Tender leaves that are 10 days old were excised and transferred to 5 ml of toxin fraction extracted from *C. cassiicola*. Disease resistance was scored from 1 to 5 based on the degree of wilting intensity of leaves caused by the toxin, where 1 indicates high level of resistance and 5 indicates high level of susceptibility. As controls, leaves from the resistant and susceptible parents *H. benthamiana* (Control 1) and *H. brasiliensis* (Control 2), respectively were placed on vials containing 5 ml of modified Czapek Dox liquid media without toxin from the pathogen

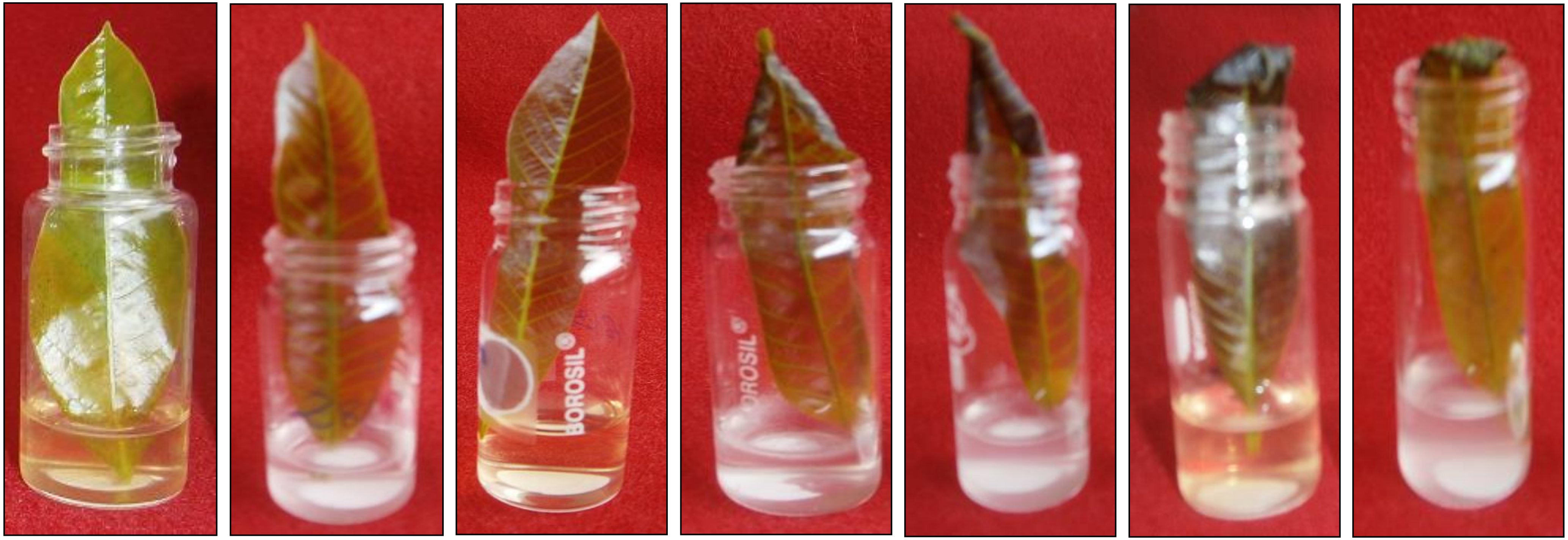

Control 1 | Control 2 | 1 | 2 | 3 | 4 | 5

Figure S6. Toxin-based screening for disease resistance to *Colletotrichum acutatum*. Tender leaves that are 10 days old were excised and transferred to 5 ml of toxin fraction extracted from *C. acutatum*. Disease resistance was scored from 1 to 5 based on the degree of wilting intensity of leaves caused by the toxin, where 1 indicates high level of resistance and 5 indicates high level of susceptibility. As controls, leaves from the resistant and susceptible parents *H. benthamiana* (Control 1) and *H. brasiliensis* (Control 2), respectively were placed on vials containing 5 ml of modified Czapek Dox liquid media without toxin from the pathogen

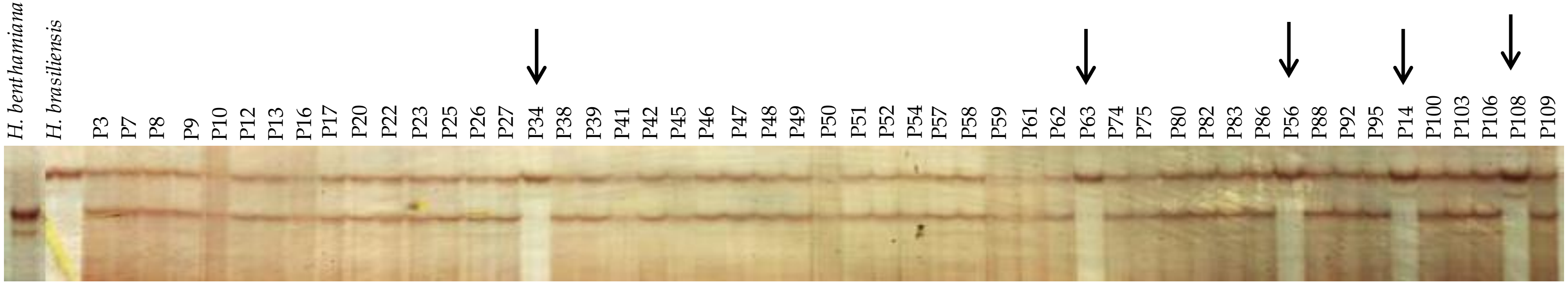

Figure S7. Representative silver stained PAGE showing allelic profiles of the interspecific mapping population including the parents *H. brasiliensis* and *H. benthamiana* using a homozygous SSR marker hmCT44, which show polymorphism between the parents. Homozygous off types (P34, P63, P56, P14 and P108) could easily be detected from the heterozygous hybrid progenies
